# The effects of fatty fish intake on adolescents’ nutritional status and associations with attention performance: results from the FINS-TEENS randomized controlled trial

**DOI:** 10.1186/s12937-018-0328-z

**Published:** 2018-02-23

**Authors:** Katina Handeland, Siv Skotheim, Valborg Baste, Ingvild E. Graff, Livar Frøyland, Øyvind Lie, Marian Kjellevold, Maria W. Markhus, Kjell M. Stormark, Jannike Øyen, Lisbeth Dahl

**Affiliations:** 1 Institute of Marine Research (IMR), P.O. Box 1870 Nordnes, 5817 Bergen, Norway; 2 Department of Clinical Medicine, Faculty of Medicine and Dentistry, University of Bergen, P.O. Box 7800, 5020 Bergen, Norway; 3 Regional Centre for Child and Youth Mental Health, Uni Research Health, P.O. Box 7810, 5020 Bergen, Norway; 4 Present Address: Uni Research Health, P.O. Box 7810, 5020 Bergen, Norway; 5 Present Address: Directorate of Fisheries, P.O. Box 185 Sentrum, 5804 Bergen, Norway; 6 Department of Health Promotion and Development, University of Bergen, P.O. Box 7807, N-5020 Bergen, Norway

**Keywords:** N-3 LCPUFA, Vitamin D, Iron, Iodine, Fatty fish, Dietary intervention, Healthy adolescents

## Abstract

**Background:**

Adolescence involves changes in dietary habits that may induce imbalances in the intake of different nutrients. Fish is an important dietary source of omega-3 (n-3) long-chain polyunsaturated fatty acids (LCPUFAs), vitamin D, several minerals and high-quality protein. By using secondary outcomes and exploratory analyses, the aims of this paper were to evaluate if nutritional biomarkers (red blood cell fatty acids, serum (s)-25(OH)D, s-ferritin and urinary iodine concentration (UIC)) were altered during a dietary intervention, and if they mediated previously reported changes in attention performance. In addition, to examine the status of the biomarkers and explore associations between dietary pattern, biomarkers and attention performance cross-sectionally at baseline.

**Methods:**

The Fish Intervention Studies-TEENS (FINS-TEENS) was a three-armed intervention trial, including adolescents from eight secondary schools (*n* = 415; age: 14–15y) in Bergen, Norway. Participants were individually randomized to receive either fish meals, meat meals or n-3 LCPUFA supplements, three times a week for a total of 12 weeks. Blood and urine samples were collected pre and post intervention and attention performance was assessed with the d2 test of attention. Analyses of covariance (ANCOVA) assessed differences between groups in changes of biomarkers and linear mixed models were applied in analyses of attention performance and biomarkers. The trial is registered in ClinicalTrials.gov (NCT02350322).

**Results:**

At baseline, the mean omega-3 index was 5.8 ± 1.3% and deficient status were identified for s-25(OH)D (54%), s-ferritin (10%) and UIC (40%). The intervention resulted in an increase in DHA and the omega-3 index which was larger in the supplement group compared to the fish and meat group (*P* < 0.01), and in the fish group compared to the meat group (*P* < 0.01). No differences between the groups were observed for changes in 25(OH)D, s-ferritin or UIC. None of the biomarkers mediated performance in the d2 test. The intake of fatty fish and a healthy dietary pattern was associated with scores in processing speed at baseline.

**Conclusions:**

These results show that Norwegian adolescents have insufficient status of important nutrients, which may be improved with fatty fish consumption or n-3 LCPUFA supplements. However, nutritional status was not associated with scores in the d2 test of attention.

## Background

Seafood is a component of dietary patterns associated with good health, and food based dietary guidelines in almost all European countries include recommendations on fish consumption. One purpose of these recommendations is to ensure the provision of key nutrients, since fish is the main dietary source of the omega-3 (n-3) long-chain polyunsaturated fatty acids (LCPUFAs) and vitamin D, in addition to minerals and high-quality protein [1, 2]. Adolescence is a critical period involving major physiological changes, and thus, suboptimal dietary habits leading to nutritional imbalances may be of special concern in this group [3]. The cross-sectional Healthy Lifestyle in Europe by Nutrition in Adolescence (HELENA) study found that 42% had a serum 25-hydroxyvitamin D (25(OH)D) concentration < 50 nmol/L [4], which is regarded as insufficient [5]. No official cut-off for the nutritional status of n-3 LCPUFAs currently exists, but a commonly used marker for dietary intake and cardiovascular disease risk is the omega-3 index, which is the percentage of eicosapentaenoic acid (EPA) and docosahexaenoic acid (DHA) in red blood cell (RBC) fatty acids [6]. The proposed omega-3 index risk categories for cardiovascular disease in adults are: high risk, < 4%; intermediate risk, 4–8%; and low risk, > 8% [7]. Based on these cut-offs, a cross-sectional study reported that only 0.4% of 1300 Australian adolescents had an omega-3 index of > 8%, 84% had an index of 4–8%, and 15.6% had an index of < 4% [8]. The food based dietary guidelines for fish consumption generally range from 100 to 300 g per week and the European food safety authority (EFSA) recommends that the dietary advice for children aged 2–18 years should be 250 mg/d of EPA and DHA [9]. In Norway, the recommendation is 2–3 dinner portions/week, which should correspond to 300–450 g/week, of which 200 g should be fatty fish. However, data from Norway and other Western populations suggest that these recommendations are not met [1, 10].

Although the brain reaches approximately 90% of its adult size at age six, it continues to undergo changes throughout adolescence, involving e.g. myelination of axons, synaptic pruning and increased communication between the brain regions [11]. Nutrition is a key environmental factor to consider in terms of brain development and function, because it can be manipulated relatively easily [12]. To our knowledge, no previous randomized controlled trials (RCT) have provided fish to adolescents and measured nutritional status and/or cognitive performance. Two observational studies have been conducted, showing that fish consumption was positively associated with adolescents’ performance at school [13] and in cognitive tests [14, 15]. Given its nutritional properties, and because increased consumption potentially could replace other foods with putative adverse health effects, it is plausible that an increased intake of fish could be more beneficial than consuming supplements. Thus, we performed a RCT to assess the effect of a 3-month dietary intervention with fatty fish meals in typically developing adolescents. Previously reported results from this study suggest that the fish meals could have led to improvements in outcomes of attention performance, although the results were difficult to interpret due to low dietary compliance [16]. By using secondary outcomes and exploratory analyses, the aims of this paper were to evaluate if nutritional biomarkers (RBC fatty acids, serum (s)-25(OH)D, s-ferritin and urinary iodine concentration (UIC)) were altered during the intervention, and if they mediated attention performance. In addition, to examine the status of nutritional biomarkers and explore associations between dietary pattern, biomarkers, and attention performance cross-sectionally at baseline.

## Methods

### Study design and ethics

Fish Intervention Studies-TEENS (FINS-TEENS) was conducted at eight lower secondary schools in Bergen, Norway, between February and May 2015. The three-armed trial used a RCT design to investigate the cognitive and nutritional effects of providing meals with fatty fish, or similar meals with meat/cheese, or fish oil supplements to adolescents.

The trial was conducted according to the declaration of Helsinki. Ethical approval was obtained from the Norwegian Data Protection Official for Research (project number: 41,030). Written informed consent was collected from all participants and one legal caregiver, and participants could withdraw from the trial without giving any reason. The trial is registered in ClinicalTrials.gov (NCT02350322). The design and methods are described in detail previously [17].

### Participants and randomization

Eligible adolescents were girls and boys attending 9th grade (14–15 years-old) at the participating schools, who knew the Norwegian language orally and written. Exclusion criteria were allergy or intolerance to the study foods or supplements. The random allocation was performed individually stratified by gender, by two researchers. One researcher went through the list of participants stating only the participants’ gender (boy/girl), while another (blinded from the list) drew lots marked with one of the three intervention groups from the correct box labeled either “boy” or “girl”.

### Study meals and n-3 supplements

The meals were prepared by a catering service (Søtt+Salt A/S, Bergen, Norway) and were similar in content except from the meat and fish. Meals in the fish group contained salmon, mackerel and herring, whereas meals in the meat group contained chicken, turkey, beef, lamb and cheese. In addition, the meals comprised vegetables and/or salad and mainly wholegrain pasta-, focaccia-, baguette- or tortilla and sometimes dressing. Halal meat and gluten free products were provided on request. The meals had a mean weight of 230 g/portion, and the amount of fish/meat was requested to be between 80 and 100 g/portion. The number of capsules was calculated to match the weekly intake of EPA and DHA in the fish group, and was seven per serving. Capsules were Nycoplus® Omega-3, 500 mg, produced by Takeda Nycomed, Asker, Norway, bought at a public pharmacy in Bergen, Norway. Fatty acids and nutrient content in the meals and supplements were analyzed and results are summarized in Table 1.

**Table 1:** Fatty acid profile and nutrient content in each portion of fish and meat meals (mean weight 230 g) and n-3 supplements

Nutrients	Fish meals	Meat meals	Supplements^d^
Total fat^a^	23.3 ± 9.1	19.3 ± 9.5	3.5
Protein^a^	24.8 ± 9.1	27.3 ± 7.7	–
LA (18:2n-6)^b^	3530.7 ± 1556.2	2753.1 ± 1453.4	35.1 ± 0.6
AA (20:4n-6)^b^	66.0 ± 36.6	51.3 ± 32.0	67.9 ± 1.1
EPA (20:5n-3)^b^	350.4 ± 456.2	7.3 ± 3.6	1079.6 ± 25.7
DPA (22:5n-3)^b^	91.7 ± 73.0	13.8 ± 6.5	119.1 ± 3.4
DHA (22:6n-3)^b^	603.2 ± 722.4	11.6 ± 12.0	743.8 ± 24.0
Sum n-3^b^	2100.6 ± 1688.1	434.6 ± 257.8	2138.5 ± 86.0
Sum n-6^b^	3738.1 ± 1557.4	2837.3 ± 1462.5	147.6 ± 1.8
Vitamin D_3_^c^	4.9 ± 2.7	< 0.1 ± 0.1	–
Iodine^c^	11.4 ± 20.3	5.9 ± 6.1	–
Selenium^c^	16.8 ± 10.7	12.1 ± 4.5	–

Abbreviations: *AA* arachidonic acid, *LA* linoleic acid, *EPA* eicosapentaenoic acid, *DPA* Docosapentaenoic acid, *DHA* docosahexaenoic acid, *n-3* omega-3, *n-6* omega-6

^a^g/portion

^b^mg/portion

^c^μg/portion

^d^1 portion = 7 capsules

Data given as mean ± SD

### Procedure

The meals and supplements were delivered three times a week for a total of 12 weeks. Participants received the meals or supplements from a study worker at their school, during the lunch break (usually between 11:00 a.m. and noon). Participants in the different intervention groups ate together in their respective class-rooms. The fish and meat meals replaced the participants’ usual lunch, whereas the supplement group continued to eat their habitual lunch in addition to taking the supplements. The school lunch of Norwegian adolescents is usually a packed lunch from home, containing medium dark or dark bread or crispbread with meat, cheese or liver pate as spread, and sometimes a fruit or vegetable [10, 18]. The participants were asked not to change any procedures they had besides the intervention, e.g. use of fish-oil supplements or their habitual dietary intake of fish at home. Dietary compliance was monitored throughout the trial by study staff who registered the remaining number of capsules and the amount of fish/meat eaten for each participant. The amount of fish/meat eaten was estimated by eye and registered on a scale from zero to four: ‘0 = nothing eaten’, ‘1 = 1/4 eaten’, ‘2 = 2/4 eaten’, ‘3 = 3/4 eaten’ and ‘4 = all eaten’.

### Questionnaire

General information about participants (age, weight, height, and gender) and their background diet (habitual dietary intake besides the intervention) were obtained with a revised and extended version of a validated web-based food frequency questionnaire (FFQ) at pre and post intervention [19, 20]. Height and weight were not measured directly in order to prioritize the cognitive tests and blood and urine sampling. The FFQ included questions about the consumption of different fish species for dinner (never – ≥4 times/week) and in the analyses, continuous indices were made from the reported intake of salmon, herring and mackerel according to the methodology by Markhus and colleagues [19], and summarized into one continuous variable reflecting fatty fish intake (range 0.0–6.0). The questionnaire included one question about physical activity (≤30 min – 4 h or more/week) and questions regarding the frequency of using solarium (never – 2 times/week) and duration of being abroad to high-UV radiation areas (range: zero – ≥4 weeks) the past three months. The reported use of solarium was dichotomized into less than once per month or ≥once per month, and the duration of being abroad to high-UV radiation areas was dichotomized into less than one week or ≥one week. These variables were combined into solarium/high-UV exposure yes/no (categorical variable). A diet score (0–8 points) which evaluates the adherence to the current Norwegian dietary recommendations by scoring the reported intake of fruits, vegetables, wholegrain, fish, red meat, dairy products, added sugar, water and physical activity has been developed and applied to the FFQ [21]. The diet score was used in the cross-sectional analyses in the present paper.

A questionnaire sent to the caregivers by e-mail assessed parental educational level (elementary/lower secondary school – college/university ≥4 years), total household income (< 200,000 NOK – > 2000,000 NOK (100 NOK = approximately 10€/11$) and origin (participant and both parents born outside or in Norway). The mean parental educational level ((mothers’ level + fathers’ level)/2) (continuous variable) was used in the statistical analyses.

### Outcomes

#### Blood and urine samples, and biochemical analyses

Authorized biomedical laboratory scientists obtained non-fasting blood samples from the elbow cavity of the participants. For preparation of RBC, venous blood was collected in BD Vacutainer® vials and centrifuged (10 min, 1000 g, 20 °C) within 30 min. RBCs were adequately separated to ensure a clean blood fraction. Venous blood for serum preparation was collected in BD Vacutainer® vials and set to coagulate for minimum 30 min before centrifuged (10 min, 1000G, 20 °C,) within 60 min. Blood samples were temporarily stored and transported on dry ice before storage in a − 80 °C freezer until analysis.

For analyzation of fatty acids, blood samples were thawed on a shaker (Nutating shaker) and prepared by the Hamilton Microlab Star Line robot. The samples were homogenized and internal standard (0.4 mg/ml Methyl Nonadecanoate in methanol) and 2% sulfuric acid in methanol were added. Samples were shaken and boiled at 105 °C for 40 min before cooled. Extraction of fatty acids was performed by adding water and heptane, and the fatty acid composition was determined on a Trace GC Ultra gas chromathograph (Thermo Corporation). The samples were analyzed on a UltraFast UFC-WAX Column (5 m × 0.1 mm × 0.1um), ThermoFischer Scientific. Helium was used as mobile phase at 0.5 ml/min constant flow. Certified reference materials were analyzed to assess the accuracy and precision of the method. A 10% difference was accepted in contents of 0.6–100% and all RBC fatty acids are expressed as weight percentage of total fatty acids. The omega-3 index was calculated as the content of EPA and DHA expressed as percent of total fatty acids [6].

S-25(OH)D concentration was determined by standardized procedures at IMR, using a liquid chromatographic-tandem mass spectrometric (LC-MS/MS) assay adding acetonitrile and internal standard (^2^H 25(OH)D_3_) to the samples [22]. S-ferritin was analyzed at Haraldsplass Diakonale Hospital Bergen, Norway, by an automated electrochemiluminescence immunoassay (ECLIA) on Cobas e601 (Roche). The urine samples was taken at home in the morning or at school and iodine status was determined as UIC in spot samples by Inductive Coupled Plasma Mass Spectrometry (ICP-MS) at IMR [23].

#### Cognitive tests

The primary outcome of the study was performance on the d2 test of attention [24]. In addition, participants completed a Norwegian reading and spelling test named “Kartleggeren” and the Strengths and Difficulties Questionnaire (SDQ), which assesses mental health status, at pre and post intervention. Results from “Kartleggeren” are not reported, due to considerable ceiling effects in nearly all outcomes both pre and post intervention. Effects of the intervention on SDQ are reported in Skotheim et al. [25]. Consequently, results from the d2 test of attention only are used in the present paper, and these have also previously been presented in more detail [16].

The d2 test of attention is a pen and paper cancellation test, measuring abilities in visual scanning, processing speed and degree of accuracy, regardless of intelligence level [24, 26]. The test comprises 47 interspersed target- and distraction characters× 14 rows, and the respondents’ task is to cancel out as many target characters as possible, while ignoring distraction characters. Standard outcomes of the test were included in the trial, which are: ‘processing speed’ (TN; total number of characters processed) and two overall measures of performance: ‘total performance’ (TN-E; total number of characters processed minus total errors made) and ‘concentration performance’ (CP; total number of correctly cancelled out target characters minus commission errors). In addition, there are two types of error-outcomes: ‘omission errors’ (E1; unmarked target characters) and ‘commission errors’ (E2; incorrectly cancelled distraction characters), and ‘total errors’ (E total; the sum of E1 and E2).

The same trained study staff administered the test according to standard instructions pre and post intervention. Testing was performed in classrooms between 9 and 11 a.m. Environmental factors such as noise were controlled for rigorously by the study crew and the teachers.

### Sample size

The sample size calculation was based on the design with a three armed-intervention, and pre and post intervention measurements with an assumed correlation of 0.5. The primary outcome of the trial was the d2 test of attention. To be able to reveal a meanigful effect of the intervention on the primary outcome, a small to moderate effect size (0.35) was applied. The calculated sample size was 119 participants in each group based on a power of 80% and a significance level of α = 0.05. When taking a 20% drop out rate into account, the aim was to enroll a sample of 446 participants in the trial.

### Statistical analyses

Data are presented as either means and standard deviations (SD) or as numbers (n) and percentages (%). At baseline, differences between intervention groups were assessed with one-way ANOVA for continuous variables and Chi-square test for categorical variables.

Paired-samples *t*-tests were used for analysis of changes in nutritonal biomarkers (fatty acids, 25(OH)D, s-ferritin or UIC) within each intervention group from pre to post intervention. ANCOVA were used to test for differences between intervention groups in changes of nutritional biomarkers. The dependent variable was the value of the relevant biomarker measured at post intervention, and the model was adjusted for the same biomarker measured at pre intervention. A second model was further adjusted for dietary compliance (amount of fish, meat or n-3 supplements eaten during the trial). Pairwise comparisons with Bonferroni correction between intervention groups were performed if the overall *P*-value was significant.

To investigate if biomarkers could mediate the assosiation between intervention and TN, TN-E and E1, which as previously reported were the significant outcomes from the d2 test of attention [16], standard mediation approach was used [27] within linear mixed effects models. The association between the nutritional biomarkers at post intervention (independent variable) and the relevant d2 test outcome measured at post intervention (dependent variable) was assessed, one at a time. School class was included as random intercept and the models were adjusted for the relevant pre intervention d2 test score and pre intervention values of the relevant nutritonal biomarker.

Association with intervention groups and reported use of solarium and being abroad to high-UV radiation areas was assessed with chi-square test.

Baseline analyses assessing the associations between d2 test-scores (dependent) and nutritional biomarkers, or the reported intake of fatty fish, or the diet score from the FFQ (independent), were performed with linear mixed effects models with school class as random intercept. Unadjusted models and models adjusted for age, sex, parental educational level and physical activity were performed for the d2 test scores.

Two-tailed *P*-values < 0.05 were considered statistically significant. The analyses were performed using the Statistical Package for the Social Sciences (SPSS® Statistics version 24, IBM Corporation, US), except for the linear mixed effect models which were performed using Stata Statistical Software: Release 14. College Station, TX: (STATACorp LP®).

## Results

### Study population

**Fig. 1:**
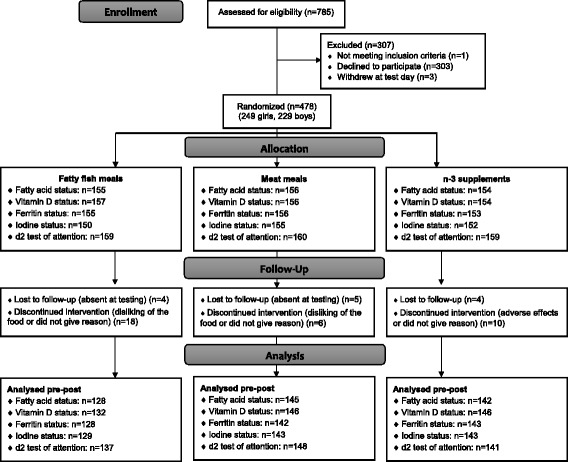
Flow chart over participants. n-3 = omega-3

Of the 785 children who were attending 9th grade at the time of recruitment and received invitation, 481 (61%) agreed to participate. Three participants withdrew from the trial at the day of baseline testing, before randomization. Thus, 478 participants were randomly allocated to intervention groups. 47 participants were lost to follow up or discontinued the intervention (*n* = 22 in the fish group, *n* = 11 in the meat group, and *n* = 14 in the supplement group) (Fig. 1). A total of 370 questionnaires (78%) were completed by the caregivers.

Participants had a mean age of 14.6 ± 0.3 years, and a mean BMI of 19.8 ± 2.9 kg/m^2^. Most were non-immigrants, defined as the participant and both of the parents were born in Norway. The participants had a mean omega-3 index of 5.8 ± 1.3% and 95% of the sample had an index ≤8%. A total of 92%, 10% and 40% had deficient levels of s-25(OH)D, s-ferritin and UIC, respectively [5, 28, 29]. The reported baseline dietary intake of fish for dinner was on average 1.5 meals/week, of which 1 of these were fatty fish. The proportion who reported to use fish oil or other types of omega-3 supplements daily was 18%, whereas 53% reported to never use this. There were no differences between the intervention groups in baseline characteristics, nutritional status or dietary intake (Table 2).

**Table 2:** Baseline characteristics of all participants and by randomly assigned intervention groups

Variables	Number	All	Fish	Meat	Supplement	*P* ^*a*^
Characteristics						
Gender n (%)	415					0.546
Male		195 (47.0)	56 (43.8)	73 (50.3)	66 (46.5)	
Female		220 (53.0)	72 (56.3)	72 (49.7)	76 (53.5)	
Age (years)	415	14.6 ± 0.3	14.6 ± 0.3	14.6 ± 0.3	14.6 ± 0.3	0.531
BMI (kg/m^2^)	385	19.8 ± 3.0	19.8 ± 3.4	19.7 ± 2.3	19.9 ± 3.1	0.940
Parental education level n (%)	341					0.793
Elementary/vocational school		132 (38.7)	41 (39.4)	47 (40.5)	44 (36.4)	
College/university		209 (61.3)	63 (60.6)	69 (59.5)	77 (63.6)	
Family income in NOK^b^ n (%)	338					0.201
< 200,000–749,999		70 (20.7)	21 (20.4)	17 (14.8)	32 (26.7)	
750,000–1,249,999		171 (50.6)	50 (48.5)	66 (57.4)	55 (45.8)	
1,250,000- > 2,000,000		97 (28.7)	32 (31.1)	32 (27.8)	33 (27.5)	
Immigrant^c^ n (%)	341	7 (2.1)	2 (1.6)	2 (1.7)	3 (2.5)	0.914
Nutritional status						
LA, 18:2n-6 (%)	415	10.8 ± 1.3	10.8 ± 1.4	10.8 ± 1.2	10.9 ± 1.3	0.707
AA, 20:4n-6 (%)	415	15.6 ± 1.5	15.6 ± 1.4	15.6 ± 1.6	15.6 ± 1.5	0.958
EPA, 20:5n-3 (%)	415	0.9 ± 0.4	0.9 ± 0.3	0.9 ± 0.4	0.9 ± 0.4	0.960
DPA, 22:5n-3 (%)	415	2.4 ± 0.3	2.4 ± 0.3	2.4 ± 0.3	2.3 ± 0.3	0.110
DHA, 22:6n-3 (%)	415	4.9 ± 1.1	4.9 ± 1.0	5.0 ± 1.2	4.8 ± 1.0	0.171
Omega-3 index^d^	415	5.8 ± 1.3	5.8 ± 1.2	5.9 ± 1.4	5.7 ± 1.3	0.304
≤ 8 n (%)		395 (95.2)	122 (95.3)	135 (93.1)	138 (97.2)	0.271
s-25(OH)D (nmol/L)	424	48.8 ± 17.4	49.1 ± 17.1	49.7 ± 17.8	48.3 ± 17.4	0.671
< 50 nmol/L n (%)^e^		229 (54.0)	72 (54.5)	76 (52.1)	81 (55.5)	0.832
s-Ferritin (μg/l)	412	40.7 ± 23.1	40.9 ± 22.4	41.2 ± 24.5	40.4 ± 22.3	0.953
< 15 μg/l n (%)^e^		40 (9.7)	12 (9.4)	13 (9.2)	15 (10.6)	0.912
UIC (μg/L)	415	122.6 ± 63.7	122.6 ± 71.3	122.9 ± 59.3	125.0 ± 66.7	0.693
< 100 μg/l^e^		164 (39.5)	55 (42.6)	49 (34.3)	60 (42.0)	0.282
Dietary intake	414					
Fish for dinner^f^		1.5 (0.9)	1.5 (1.0)	1.4 (0.9)	1.5 (0.9)	0.780
Herring/mackerel/salmon for dinner^f^		1.0 (0.9)	1.0 (1.0)	0.9 (0.9)	1.0 (1.0)	0.852
Fish as bread spread^f^		0.6 (1.0)	0.7 (1.0)	0.6 (1.0)	0.6 (0.9)	0.620
Fish oil supplements (n (%))^g^	413					0.659
Never		220 (53.3)	60 (46.9)	81 (56.3)	79 (56.0)	
1–3 times/month		53 (12.8)	18 (14.1)	18 (12.5)	17 (12.1)	
1–3 times/week		45 (10.9)	19 (14.8)	14 (9.7)	12 (8.5)	
4–6 times/week		20 (4.8)	6 (4.7)	5 (3.5)	9 (6.4)	
Every day		75 (18.2)	25 (19.5)	26 (18.1)	24 (17.0)	

Abbreviations: *AA* arachidonic acid, *LA* linoleic acid, *EPA* eicosapentaenoic acid, *DPA* Docosapentaenoic acid, *DHA* docosahexaenoic acid, *25(OH)D* 25-hydroxyvitamin D, *s-Ferritin* serum ferritin, *UIC* urinary iodine concentration, *NOK* Norwegian kroner

^a^One-way ANOVA test (continuous variables) and Pearson’s Chi-square test (*X*^2^) (categorical variables) for comparison between treatment groups

^b^100 NOK = approximately 10€/11$

^c^Immigrant was defined as participants who’s both parents and themselves were born outside Norway

^d^The content of EPA and DHA expressed as percent of total fatty acids [6]

^e^Adolescents were classified with vitamin D deficiency if s-25(OH)D < 50 nmol/L [5], with depleted iron stores if s-ferritin < 15 μg/L [29] and as iodine deficient if UIC < 100 μg/L [28]

^f^Reported meals per week (besides the intervention)

^g^N (%) of participants reporting to consume fish oil as dietary supplements

Data are given as mean ± SD if not other is indicated

A mean of 30 ± 6 meals were served to each participant during the intervention. As reported previously [17], the total intake (dietary compliance) was higher in the supplement group than in the meal groups, and the total intake of meat in the meat group was higher than the total intake of fish in the fish group. The proportion of participants who consumed at least half of the fish/meat/capsules during the trial was 38%, 66% and 87% in the fish, meat and supplement group, respectively [17]. There was not observed any differences between intervention groups in changes of the background diet from pre to post intervention [16].

### Change in nutritional status during the intervention

As shown in Table 3, the pairwise comparisons (with Bonferroni correction) showed that the omega-3 index and DHA increased in the supplement group compared to the fish and meat group, and that these markers also increased more in the fish group compared to the meat group. When adjusting for dietary compliance, these differences between groups were still significant for the omega-3 index, but the increase in DHA was no longer different between the supplement and fish group. The supplement group showed a decrease in AA during the intervention, compared to the two other groups. The fish group showed a mean increase in s-25(OH)D concentration of 5.3 nmol/L, whereas the increase in the meat and supplement group was 1.8 and 2.6 nmol/L respectively, and the initial analyses showed no differences between the groups. When adusting for dietary compliance, an overall difference was observed between the groups (*P* = 0.032), and the pairwise comparisons between the fish and meat group and the fish and supplement group gave *P* = 0.056 and *P* = 0.060, respectively. There were no associations between the intervention groups and the reported use of solarium and stays abroad in high-UV radiation areas during the trial. No differences between intervention groups were observed for changes in iron or iodine status from pre to post intervention.

**Table 3:** Changes in the adolescents’ fatty acids levels (% of total fatty acids), serum 25(OH)D, serum ferritin and urinary iodine concentration during the intervention

Variables	Fish			Meat			Supplement				
	Post	Change	*P* ^a^	Post	Change	*P* ^a^	Post	Change	*P* ^a^	*P* ^b^	*P* ^c^
LA, 18:2n-6 (%)	10.8 ± 1.4	0.0 ± 1.3	0.90	10.9 ± 1.2	0.1 ± 1.4	0.58	10.9 ± 1.3	− 0.1 ± 1.4	0.64	0.80	0.77
AA, 20:4n-6 (%)	13.4 ± 1.4	−2.2 ± 1.6	< 0.001	13.7 ± 1.3	− 1.9 ± 1.6	< 0.001	12.8 ± 1.4	− 2.8 ± 1.7	< 0.001	< 0.001^d,f^	< 0.001^d,f^
EPA, 20:5n-3 (%)	0.8 ± 0.3	− 0.1 ± 0.2	0.002	0.8 ± 0.4	− 0.1 ± 0.3	< 0.001	1.3 ± 0.5	0.5 ± 0.4	< 0.001	< 0.001^d,f^	< 0.001^d,e,f^
DPA, 22:5n-3 (%)	2.0 ± 0.3	− 0.3 ± 0.3	< 0.001	2.0 ± 0.3	− 0.4 ± 0.3	< 0.001	2.3 ± 0.3	0.0 ± 0.4	0.59	< 0.001^d,f^	< 0.001^d,f^
DHA, 22:6n-3 (%)	5.6 ± 1.0	0.6 ± 0.8	< 0.001	5.3 ± 1.1	0.3 ± 0.8	< 0.001	6.0 ± 0.8	1.2 ± 0.9	< 0.001	< 0.001^d,e,f^	< 0.001^e,f^
Omega-3 Index^g^	6.4 ± 1.2	0.6 ± 0.9	< 0.001	6.1 ± 1.3	0.2 ± 1.0	0.016	7.3 ± 1.2	1.6 ± 1.1	< 0.001	< 0.001^d,e,f^	< 0.001^d,e,f^
s-25(OH)D (nmol/L)	54.4 ± 16.7	5.3 ± 14.9	< 0.001	51.6 ± 17.3	1.8 ± 12.2	0.07	50.9 ± 18.4	2.6 ± 11.7	0.009	0.07	0.032
s-Ferritin (μg/l)	39.9 ± 21.4	− 0.7 ± 18.1	0.66	38.1 ± 22.7	− 3.0 ± 14.8	0.017	37.0 ± 23.9	−3.3 ± 18.7	0.040	0.40	0.58
UIC (μg/L)	107.7 ± 72.2	− 11.6 ± 66.9	0.47	125.3 ± 70.3	2.4 ± 73.7	0.70	125.6 ± 65.8	0.9 ± 76.0	0.89	0.053	0.08

Abbreviations: *AA* arachidonic acid, *LA* linoleic acid, *EPA* eicosapentaenoic acid, *DPA* Docosapentaenoic acid, *DHA* docosahexaenoic acid, *s-25(OH)D* serum 25-hydroxyvitamin D, *s-Ferritin* serum ferritin, *UIC*; urinary iodine concentration

Total numbers may vary between variables due to varying numbers of missing data

^a^*P*-value for comparison within intervention groups, paired samples *t*-test

^b^*P*-value for comparison between intervention groups. ANCOVA (adjusted for the current outcome at baseline)

^c^*P*-value for comparison between intervention groups. ANCOVA (adjusted for the current outcome at baseline and dietary compliance (i.e. the total intake of fish, meat or supplements)) during the trial

^d^*p* < 0.01 fish - supplement, ^e^ *p* < 0.01 fish - meat, ^f^ *p* < 0.01 supplement - meat (Bonferroni correction for post-hoc comparison between intervention groups)

^e^The content of EPA and DHA expressed as percent of total fatty acids [6]

Data given as mean ± SD

### Associations between biomarkers of nutritional status and attention performance

None of the nutritional biomarkers were found to be associated with the post intervention value of TN, TN-E or E1, and could thereby not alone have mediated the previously reported change in these outcomes (results not shown).

Neither the unadjusted nor the adjusted cross-sectional analyses at baseline measurements showed associations between the nutritional biomarkers and performance in d2 test outcomes. An association between the participants’ diet score (0–8 points reflecting low-high adherence to dietary recommendations) and CP, TN-E and TN was found in the unadjusted analyses. This association remained significant for TN after adjusting for potential confounders. An association between the reported consumption of salmon, mackerel and herring and TN was observed in the unadjusted and the adjusted analyses (Table 4).

**Table 4:** Associations between adolescents’ cognitive test-performances and nutritional variables or FFQ data at baseline

	Unadjusted^a^			Adjusted^b^		
	Number	ß (95% CI)	P	Number	ß (95% CI)	P
Omega-3 Index^c^	460			367		
Concentration performance (CP)		0.62 (−1.75, 2.99)	0.61		−1.43 (−4.07, 1.20)	0.29
Total performance (TN-E)		−0.09 (−5.28, 5.11)	0.97		−3.56 (−9.42, 2.31)	0.23
Processing speed (TN)		−0.67 (− 6.36, 5.02)	0.82		−2.93 (−9.36, 3.51)	0.37
s-25(OH)D	462			368		
Concentration performance (CP)		−0.04 (−0.21, 0.14)	0.69		−0.08 (− 0.28, 0.11)	0.41
Total performance (TN-E)		−0.11 (− 0.50, 0.27)	0.56		−0.18 (0.62, 0.26)	0.43
Processing speed (TN)		−0.17 (− 0.58, 0.25)	0.44		−0.18 (0.66, 0.31)	0.47
s-Ferritin	459			365		
Concentration performance (CP)		0.07 (−0.06, 0.20)	0.30		0.08 (−0.08, 0.22)	0.32
Total performance (TN-E)		0.13 (−0.17, 0.42)	0.40		0.15 (−0.19, 0.49)	0.38
Processing speed (TN)		0.06 (−0.23, 0.40)	0.60		0.13 (−0.24, 0.50)	0.50
UIC	452			359		
Concentration performance (CP)		0.04 (−0.01, 0.08)	0.13		0.01 (−0.04, 0.06)	0.68
Total performance (TN-E)		0.03 (−0.08, 0.13)	0.63		−0.02 (− 0.14, 0.09)	0.70
Processing speed (TN)		−0.02 (− 0.13, 0.09)	0.75		−0.05 (− 0.18, 0.07)	0.39
Diet score^d^	465			368		
Concentration performance (CP)		2.61 (0.54, 4.69)	0.014		1.09 (−1.31, 3.48)	0.38^e^
Total performance (TN-E)		5.76 (1.35, 10.27)	0.012		4.32 (−1.00, 9.64)	0.11^e^
Processing speed (TN)		5.54 (0.61, 10.48)	0.028		6.05 (0.24, 11.85)	0.04^e^
Fatty fish consumption^f^	463			368		
Concentration performance (CP)		1.51 (−1.78, 4.81)	0.37		1.92 (−1.67, 5.52)	0.29
Total performance (TN-E)		5.86 (−1.23, 12.95)	0.11		6.98 (−0.98, 14.93	0.09
Processing speed (TN)		8.51 (0.80, 16.23)	0.031		10.08 (1.42, 18.74)	0.023

Abbreviations: *FFQ* food frequency questionnaire, *25(OH)D* 25-hydroxyvitamin D, *s-Ferritin* serum ferritin, *UIC* urinary iodine concentration

^a^Linear mixed effects model with school class as random intercept

^b^Linear mixed effects model with school class as random intercept, adjusted for age, sex, parental educational level and physical activity level

^c^The content of EPA and DHA expressed as percent of total fatty acids [6]

^d^A score including the reported intake of fruits, vegetables, wholegrain, fish, red meat, dairy products, added sugar, water and physical activity according to the Norwegian dietary recommendations. Range: 0–8 points [21]

^e^Not adjusted for physical activity

^f^Reported frequency of eating salmon, herring and mackerel from the FFQ

## Discussion

This trial among school-aged adolescents showed that a 3-month dietary intervention with n-3 LCPUFA supplements or fatty fish meals increased RBC concentrations of DHA and the omega-3 index more than meat meals. No differences between groups were observed in changes of s-25(OH)D, s-ferritin or UIC concentrations from pre to post intervention. No associations between changes in nutritional biomarkers and attention performance was observed, but the cross-sectional analyses showed an association between the participants’ healthy diet score and processing speed (TN) at baseline, and between the reported intake of fatty fish (salmon mackerel and herring) and TN at baseline. The proportion of participants with deficient levels were 54% for s-25(OH)D, 10% for s-ferritin and 40% for UIC.

The average omega-3 index at baseline was 5.8% in this sample of adolescents, which is slightly higher than the mean index of 4.9% found in 1300 Australian adolescents [8]. However, when compared with the categories for cardiovascular disease risk for adults [7], a larger proportion in our sample had an omega-3 index < 4% (5% in our sample compared to 0.4% in the Australian sample) and a smaller proportion had an omega-3 index > 8% (5% in our sample compared to 15.6% in the Australian sample). The mean increases in the omega-3 index of 0.6% and 1.6% in the fish and supplement group respectively, is comparable to the findings by Stonehouse et al.*,* who reported increases between 0.8–1.8% with salmon meals (710 mg/d EPA + DHA) or salmon oil supplements (210–600 mg/d EPA+ DHA) to healthy adults for 8 weeks [30]. The proportion with vitamin D deficiency is slightly higher in this study than among European adolescents in the HELENA study [4]. This is probably because this trial started in February, which is when s-25(OH)D concentrations are almost at their lowest in high latitudes such as Bergen, Norway, (60.4°N) due to absent UV-B radiation [31]. The mean increase of 5.3 nmol/L in the fish group is nearly comparable with the results from a meta-analysis of RCTs in adults (> 18 years), showing that the average increase in fish groups relative to placebo was 4.4 nmol/L, and when only fatty fish interventions was considered, the difference increased to 6.8 nmol/L [32]. Mostly white meat was served to the meat group, which may explain the decreased level of s-ferritin in this group, although no differences between groups were evident. We did not measure puberty stage, but it is unlikely that the onset of menarche should have affected s-ferritin levels during the relatively short duration of 12 weeks. The median UIC in this sample of adolescents was 111.8 μg/L, which is just above the epidemiologic criteria for sufficient iodine status in populations given by WHO [28]. However, it is considerably lower than the median of 200 μg/L found in Icelandic adolescent girls (*n* = 112, 16–20 y) [33]. This could be because Iceland in the past has been known for its high iodine status due to a high intake of fish and milk. The large percentage with UIC below 100 μg/L in the present trial give cause for concern, and some of the explanation might be that iodized salt is not commonly used in households, and not used at all in the Norwegian food industry. The reason why no change in UIC was observed in the fish group from pre to post intervention is possibly because we used fatty fish, whereas lean fish is usually higher in iodine contents [34].

As mentioned, this is to our knowledge the first study that has provided fish to adolescents to measure the effect on attention performance. Associations between the d2 test of attention and RBC fatty acid status (the omega-3 index) have been investigated in two other studies, one RCT in healthy school children [35] and one cross-sectional study in healthy adolescents [36]. They reported contradictory (a positive [35] and a negative [36]) associations between the omega-3 index and errors of omission (E1 errors). The RCT also found associations between the omega-3 index and scores in concentration performance (CP) and total performance (TN-E) at baseline, but not in the correlation analyses between the intervention-induced changes. Thus, the results from previous studies using the d2 test of attention are inconclusive, which brings into question whether the test assesses performance in brain areas affected by n-3 LCPUFA status. The cross-sectional analyses in the present study suggesting that there is an association between TN and fatty fish intake, and a healthier dietary pattern is interesting. Other studies in healthy young participants have reported associations between cognitive outcomes and fish intake [13, 15] and between a healthy diet or intake of healthy foods [37–40]. However, because the present results are based on cross-sectional analyses, we cannot make statements on causality; although it is tempting to make the interpretation that TN was driven by the diet measures, the opposite may be true, such that higher scores in TN may implicate better dietary choices. In future studies, a more sensitive measure of processing speed e.g. response times in milliseconds may be useful to assess the potential association between fish or dietary intake and processing speed.

Given a 100% dietary compliance rate, the consumption of three portions/week of the fatty fish meals would have provided the participants in this trial with a weekly mean dose of approximately 1050 mg EPA and 1800 mg DHA. Divided by seven this corresponds to a daily mean dose of 150 mg EPA and 257 mg DHA. This dose is lower compared to other RCTs that used supplements [41–43], but slightly higher than the daily recommendation of 250 mg/d EPA and DHA advised by EFSA [9]. However, the relatively low dietary compliance in the fish group means that these participants were exposed to a lower dose, which is probably why we observed a greater mean increase of DHA and the omega-3 index in the supplement group than in the fish group. It is possible that the dose of n-3 LCPUFAs used in the present trial was too low and the duration too short for the intervention to result in brain modifications measurable with a cognitive test. However, a RCT in healthy boys found that supplementing with both 400 mg/d DHA (which is comparable to the dose used in our study) and 1200 mg/d DHA for only eight weeks increased the amount of DHA in RBC, and that this correlated with prefrontal cortex activation as measured with fMRI [44]. The low proportion of participants found to be iron deficient may explain why we did not see any association between iron and performance in the d2 test of attention.

### Strengths and limitations

Strengths of this trial include the use of RBC fatty acids as biomarker, since this has shown to be a valid and objective indicator of fatty acid intake the prior weeks, and it is not altered in the fed state [45, 46]. Furthermore, the use of LC-MS/MS method for determination of s-25(OH)D and ICP-MS for determination of UIC is a strength because these are considered methods of high trueness and precision [47, 48]. The fact that the whole meal was homogenized and analyzed for its nutrients content after cooking gives a more accurate assessment of actual nutrient intakes by the participants compared to analyses of the plain or raw filet. In addition, the close follow-up of the participants throughout the dietary intervention provided us with useful data on dietary compliance. An important limitation of this trial was the low study dietary compliance particularly observed in the fish group. One of the main reasons for this is perhaps that we had to serve the meals cold due to practical reasons. Thus, future studies should emphasize that meals have high quality and tastiness in order to achieve acceptable compliance. Another limitation is the ceiling effect in the reading and writing test that left us with only one test on cognitive performance. A test-battery measuring other cognitive functions than just attention could have potentially revealed associations that were undetected in this trial. Furthermore, the EPA and DHA content was not equal between the fish and supplement group, although that was the aim. Paradoxically, the fish meals also had a slightly higher total content of n-6 PUFAs compared to meat, which was mainly due to a high content of LA in the farmed salmon. It could be argued that a 12 weeks duration was not sufficient, since RBC n-3 fatty acids do not plateau until around 6 months [49]. However, it has been noticed in supplementation studies that the greatest increase is achieved within the first 4–8 weeks [49, 50]. Still, it is questionable whether the duration was sufficient to achieve a clinically meaningful change in the brain that could be fully translated into performance in a cognitive test.

## Conclusions

In conclusion, the intake of n-3 supplements and fatty fish meals increased RBC levels of DHA and the omega-3 index more than meat meals, but n-3 supplements also increased more in these parameters than the fish meals, possibly due to differences in dietary compliance. None of the nutritional biomarkers were found to be associated with performance in the d2 test of attention. Baseline measurements showed deficient levels of s-25(OH)D and UIC in addition to suboptimal levels of EPA + DHA in RBC (based on adult cut-offs) in a considerable number of participants. The longer-term implications for the suboptimal nutritional status found in these adolescents is unknown, however, they give cause for concern and should be given increased attention in future studies.

## Abbreviations

25(OH)D: Serum 25-hydroxyvitamin D
AA: Arachidonic acid
CP: Concentration performance
DHA: Docosahexaenoic acid
DPA: Docosapentaenoic acid
E total: Total errors
E1: Errors of omission
E2: Errors of commission
EPA: Eicosapentaenoic acid
FFQ: Food frequency questionnaire
FINS: Fish intervention studies
LA: Linoleic acid
LCPUFA: Long-chain polyunsaturated fatty acids
n-3: Omega-3
RBC: Red blood cells
RCT: Randomized controlled trial
s-Ferritin: Serum ferritin
TN: Processing speed
TN-E: Total performance
UIC: Urinary iodine concentration
